# Dosimetric accuracy of the cone‐beam CT‐based treatment planning of the Vero system: a phantom study

**DOI:** 10.1120/jacmp.v17i4.6194

**Published:** 2016-07-08

**Authors:** Indra Yohannes, Heru Prasetio, Karoline Kallis, Christoph Bert

**Affiliations:** ^1^ University Hospital Erlangen Radiation Oncology Erlangen Germany; ^2^ Friedrich‐Alexander‐Universität Erlangen‐Nürnberg Erlangen Germany

**Keywords:** HLUT, Vero CBCT system, dose calculation

## Abstract

We report an investigation on the accuracy of dose calculation based on the cone‐beam computed tomography (CBCT) images of the nonbowtie filter kV imaging system of the Vero linear accelerator. Different sets of materials and tube voltages were employed to generate the Hounsfield unit lookup tables (HLUTs) for both CBCT and fan‐beam CT (FBCT) systems. The HLUTs were then implemented for the dose calculation in a treatment planning system (TPS). Dosimetric evaluation was carried out on an in‐house‐developed cube phantom that consists of water‐equivalent slabs and inhomogeneity inserts. Two independent dosimeters positioned in the cube phantom were used in this study for point‐dose and two‐dimensional (2D) dose distribution measurements. The differences of HLUTs from various materials and tube voltages in both CT systems resulted in differences in dose calculation accuracy. We found that the higher the tube voltage used to obtain CT images, the better the point‐dose calculation and the gamma passing rate of the 2D dose distribution agree to the values determined in the TPS. Moreover, the insert materials that are not tissue‐equivalent led to higher dose‐calculation inaccuracy. There were negligible differences in dosimetric evaluation between the CBCT‐ and FBCT‐based treatment planning if the HLUTs were generated using the tissue‐equivalent materials. In this study, the CBCT images of the Vero system from a complex inhomogeneity phantom can be applied for the TPS dose calculation if the system is calibrated using tissue‐equivalent materials scanned at high tube voltage (i.e., 120 kV).

PACS number(s): 87.55.de, 87.56.Fc, 87.57.qp

## I. INTRODUCTION

Cone‐beam computed tomography (CBCT) using the kilovoltage (kV) imager installed on modern linear accelerators offers the possibility to improve accuracy of patient positioning before each treatment. In addition to the anatomical information of the patient, the acquired CBCT images provide a quantitative estimate of the attenuation (i.e., the Hounsfield unit (HU)) that can be used for dose calculation.^1^, ^2^, ^3^, ^4^, ^5^, ^6^, ^7^ However, due to scatter and artifacts in CBCT images that are more than those in fan‐beam CT (FBCT), the accuracy of the CBCT‐based dose calculation should be carefully assessed.^5^, ^6^, ^8^, ^9^ Moreover, several authors have reported that the accuracy of the dose calculated from CBCT images is reduced when the effects of inhomogeneities are incorporated.^1^, ^10^

The dose calculation in a treatment planning system (TPS) is based on the conversion of HU to mass density or electron density in order to take into account the tissue inhomogeneity. This conversion is represented by a HU lookup table (HLUT). The parameter that most affects the accuracy of the HLUT is the applied tube voltage (kV) during image acquisition.^4^, ^11^ Furthermore, the use of the bowtie filter improves the image quality and hence the accuracy of the HLUT.^1^, ^5^, ^12^ The HLUT is determined in phantom‐based measurements in which a number of known materials are imaged such that the measured HU can be linked to the known electron density. Typically, a phantom with multiple inserts of these tissue‐equivalent materials (TEM) is used for the calibration measurement.^5^, ^6^ Several studies have also investigated the impact of the phantom insert materials on the HLUT accuracy either in FBCT or in CBCT.^5^, ^6^, ^13^, ^14^ These studies have shown that the use of materials that are not tissue‐equivalent can cause dose calculation errors.

A real‐time tumor tracking system which uses a gimbaled linac, the Vero system (Brainlab AG, Feldkirchen, Germany),^(15)^ is equipped with a stereoscopic dual‐source kV X‐ray imaging system for patient positioning and image guidance for tracking.^(16)^ In combination with the FBCT images and the treatment plan optimized based on the FBCT, the CBCT data obtained using this kV imaging system can, in principle, be used for adaptive radiotherapy purposes (i.e., treatment plan adaptation in reaction to potential changes in patient anatomy).^2^, ^5^, ^6^ However, the accuracy of the dose calculated directly from the CBCT images of this nonbowtie filter kV imaging system has not yet been investigated. Thus, the aim of this study is to evaluate the dosimetric accuracy of CBCT‐based treatment planning for the Vero system. Dosimetric results were compared to evaluate the CBCT‐based and FBCT‐based plans using different parameters (i.e., tube voltages and phantom insert materials) with the measurements in a phantom.

## II. MATERIALS AND METHODS

### A. Cone‐beam CT system

**Figure 1:**
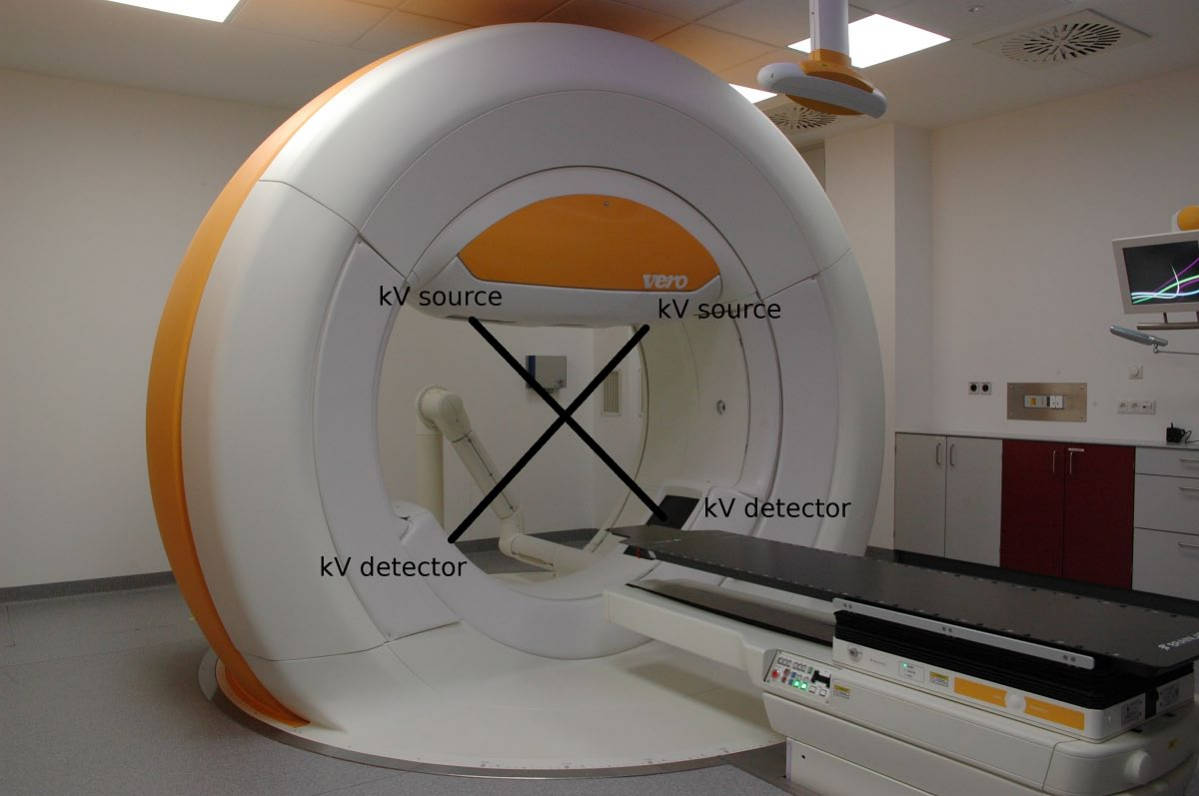
A Vero system equipped with two kV imagers for CBCT.

Investigation of the accuracy of the CBCT‐based dose calculation was performed on the Vero system (see Fig. 1). The imaging system of the Vero consists of two identical X‐ray tubes (Shimadzu Corp., Kyoto, Japan) and two amorphous silicon detectors (PaxScan 4030A; Varian Medical Systems, Palo Alto, CA). The distance between the X‐ray tube and the detector is 187.6 cm. The system does not have a bowtie filter. The imaging system acquires one projection image for every 0.5° either with clockwise rotation from 320° to 175° or counterclockwise rotation from 40° to 185°.^(17)^ In this work, we used only one kV imager with clockwise rotation to obtain CBCT datasets. The volume images were reconstructed with the Shimadzu algorithm. The maximum field of view (FOV) is limited to 20 cm in diameter and 15 cm in length.

### B. Image acquisition for HU lookup table

We scanned four different sets of materials to obtain the HU lookup tables (HLUT). The FBCT used the Somatom Sensation Open (Siemens Healthcare GmbH, Forchheim, Germany) for treatment planning, and the CBCT used the Vero's system. The CTP404 module in the Catphan 504 phantom (The Phantom Laboratory, Salem, NY), the Gammex 467 tissue characterization phantom (Gammex Inc., Middleton, WI), a set of tissue‐ and water‐equivalent materials developed using the stoichiometric analysis method (SAM) by Yohannes et al.^(14)^ (QRM GmbH, Möhrendorf, Germany), and a set of materials (see Table 1 from Yohannes et al.^(18)^) for stoichiometric calibration (SC) were utilized in this work. The diameters of the inserts are 1 cm, 2.8 cm, and 2 cm for the CTP404, the Gammex 467, and all other materials, respectively. Except for the Catphan phantom (20 cm diameter) that already provides the volumetric scatter, each of the other materials was scanned in the middle of a 20 cm diameter by 16 cm length water phantom to give the volumetric scatter to the materials.^(6)^ Three different tube voltages (i.e., 80, 100, and 120 kV) and a 3 mm slice thickness for image reconstruction were employed in both CT systems to assess the impact of this scan parameter on dose calculation accuracy. We applied 200 mAs and 100 mA for acquiring FBCT and CBCT images, respectively. The difference of mA should, in principle, not affect the dose calculation.^(11)^ Subsequently, the mean HU inside each material insert was determined and plotted against its mass density to obtain HU‐density LUT as input for Pinnacle^3^ TPS (Philips Radiation Oncology Systems, Fitchburg, WI).

### C. Dosimetry measurements

**Figure 2:**
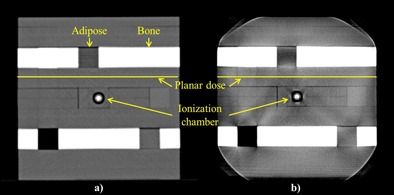
An in‐house phantom used for dosimetry study scanned in (a) FBCT and (b) CBCT with tube voltage of 120 kV.

Dosimetric evaluation was performed on an in‐house cube phantom (edge length: 16 cm). The phantom consists of water‐equivalent slabs and several inserts that ranged from air to bony structures and has an adapter for an ionization chamber to measure the absolute dose at that particular location (see Fig. 2). The cube phantom was designed so that the inserts of adipose $\left(\rho =0.95\;g/{\text{cm}}^{3}\right)$ and bone $\left(\rho =1.60\;g/{\text{cm}}^{3}\right)$ were positioned above the planar dose location to represent both the soft‐tissue and bone structures of the HLUTs. The planar dose was measured at 6 cm below the surface of the phantom. Furthermore, measurements using 6 MV X‐ray beams with 200 MU from the Vero system were done. We applied a single anterior–posterior field of 10 cm × 10 cm with the isocenter at the middle of the cube phantom to investigate the effects of HLUTs from different materials and tube voltages of both CT systems in the dose calculation. Two independent dosimeters at two different locations inside the cube phantom were used in this investigation for point‐dose and two‐dimensional (2D) dose distribution measurements. The PTW 23332 ionization chamber (PTW, Freiburg, Germany), which was connected to an electrometer (Dose 1, IBA Dosimetry GmbH, Schwarzenbruck, Germany), was used to measure the point‐dose, while the 2D dose distribution measurements were done using EDR2 film (Carestream Health Inc., Rochester, NY).

For calibration purposes, the EDR2 films were placed perpendicular to the beam axis 5 cm deep in RW3 slabs (PTW) with an additional 5 cm RW3 as backscatter material. Each of the calibration films was exposed with a 10 cm×10 cm field size at a 100 cm source‐to‐surface distance that covered a dose range between 20 and 470 cGy. The film was then scanned using a Vidar VXR Dosimetry‐Pro 16‐bit scanner (Vidar Systems Corp., Herndon, VA) to obtain a calibration curve of the film using the relation between the dose and image pixel value (PV).^(19)^ By applying the resulting calibration curve, the film's planar absolute dose distribution in the cube phantom of the irradiated field was acquired.

### D. Dose calculation in TPS and evaluation

The HLUTs from different sets of materials and tube voltages were first recorded in the TPS. We scanned also the cube phantom, with a PTW 23332 ionization chamber inserted in the phantom, both in FBCT and CBCT with the same settings (i.e., kV, slice thickness, and mA/mAs), as described in the section B above. A volume of interest of the ionization chamber was drawn to obtain the calculated dose in that area. In addition to the dose determined by the ionization chamber, we calculated the planar dose at 6 cm below the surface of the phantom. The gamma evaluation method^(20)^ with criteria of 3% delta dose and 3 mm distance to agreement (DTA) was performed with the OmniPro I'mRT software (IBA Dosimetry GmbH) to evaluate the planar absolute dose maps from the TPS with the measured one from the film. All calculations in this work were performed using the collapsed cone convolution (CCC) algorithm in Pinnacle^3^. Each plan of the cube phantom from every CT system and tube voltage was calculated using the HLUTs of different sets of materials obtained with the same CT system and tube voltage. All planning parameters were kept the same for all CBCT and FBCT images of the phantom by using an in‐house Pinnacle^3^ script.

## III. RESULTS AND DISCUSSION

**Figure 3:**
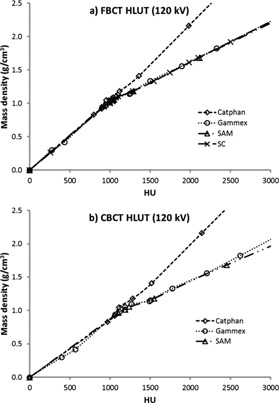
HLUT from different sets of materials resulting from (a) FBCT and (b) CBCT at 120 kV.

Figure 3 shows the HLUTs from all sets of materials both in FBCT and CBCT that were used for the dose calculation in Pinnacle^3^. Only the HLUTs at 120 kV are presented in Fig. 3, since similar patterns were measured at the other tube voltages. The HLUTs from the SC were plotted using the ICRU tissue database^21^, ^22^, summarized in Table 2 of Yohannes et al.^(14)^ Although the HLUT of the SC for the FBCT is well established, the approach used in the SC failed to calibrate the CBCT due to its volumetric scattering, which differs from the FBCT. Thus, only the HLUT of the SC for the FBCT is shown in Fig. 3. In general, the HLUTs of the Gammex, SAM, and SC were similar, since they are either tissue‐equivalent‐based materials (Gammex and SAM) or a tissue‐equivalent calibration technique (SC). The HLUTs of the SAM materials were in good agreement with the SC as shown in Fig. 3(a) since those materials were developed in an approach that is based on the SC.^(14)^ In contrast to the other materials, the HLUTs from the Catphan phantom show differences especially in the high‐density inserts since the bones were represented by Delrin and Teflon that are not tissue‐equivalents.^4^, ^5^, ^6^

**Figure 4:**
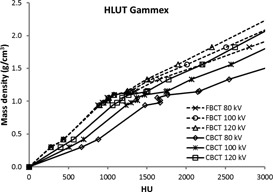
HLUTs of the Gammex with solid lines taken from CBCT and dashed lines taken from FBCT for all tube voltages.

Moreover, the tube voltage used to obtain the CT images plays an important role in the image quality. This impact is even greater in the CBCT system, as shown in Fig. 4 (only the HLUTs of Gammex are depicted, since similar shapes were determined for the other materials). It was observed that as the tube voltage decreases, the HU increase and the HU differences of the same inserts resulting from the different tube voltages rise steadily as the mass density increases. The maximum difference of 1,110 HU between the CBCT images at 80 kV and 120 kV was detected for the highest‐density insert of the SAM materials $\left(\rho =1.68\;g/{\text{cm}}^{3}\right)$. A lower tube voltage used for CT scanning led to a higher HU, due to the increased photoelectric attenuation, especially in high‐density inserts (bony structures). The shift of HU is larger in CBCT because of the volumetric scatter that generates more attenuation in the lower kV. Furthermore, the lack of the bowtie filter in the Vero's CBCT system may introduce more scatter and low energy photons, which can cause higher HU.^1^, ^12^

The differences of HLUTs from various tube voltages and inserts in both CT systems result in the differences of the dose calculation accuracy, as shown in Tables 1 and 2 for point‐dose and 2D dose distribution of the cube phantom, respectively. As presented in Tables 1 and 2, the higher kV, the better point‐dose calculation and gamma passing rate. This is due to the fact that the Compton scatter dominates in 6 MV beams. The Compton process is almost independent of atomic number and proportional to density. In contrast to the high‐energy beams used to acquire the measured dose, the photoelectric effect is the predominant process in the low tube voltage used for obtaining the HLUTs, in particular 80 kV, and in high atomic number materials. This effect in bones will cause higher attenuation at lower kV and consequently deposit more doses in the calculation. The point‐dose measurement results do not show this difference extensively (see Table 1) since the chamber was in the middle of adipose and bone (beam's‐eye‐view perspective). Nevertheless, from the 2D dose maps comparison, the differences of the tube voltages and the inserts affected the gamma analysis results (see Table 2). As expected from the HLUTs, the Pinnacle^3^ calculated lower dose in the chamber for CBCT due to the higher HU compared to the FBCT. Moreover, the composition of the Catphan material inserts that are not tissue‐equivalent leads to higher dose calculation inaccuracy compared to the other materials, and this effect is greater in CBCT. Additionally, it is worth noting that even with a good point‐dose calculation, there is no guarantee that the planar dose calculated using the same HLUT will give a good passing rate as well. Therefore, we recommend that the evaluation of the HLUTs dose accuracy is done at least using the 2D dose maps. Further, Van Dyk et al.^(23)^ and Ahnesjö et al.^(24)^ proposed the accuracy criteria for photon beam dose calculations using inhomogeneity phantoms of 4% in high‐dose region/low‐dose gradient and 4 mm in large dose gradient. Based on their recommendations and in view of the fact that we utilized a complex heterogeneous phantom, we even implemented an optimistic 3% dose difference and 3 mm DTA as the gamma parameter. In this investigation, the Vero's CBCT images can be used for the dose calculation if they are calibrated using either the Gammex or the SAM materials, but only at 120 k V.

**Table 1:** Calculated chamber doses and their relative differences to the measured dose resulting from different HLUTs at different tube voltages both in FBCT and CBCT

	*80 kV*		*100 kV*		*120 kV*	
*Measured Chamber Dose:* 1.680±0.015 Gy	*Calculated Chamber Dose (Gy)*	Δ *(%)*	*Calculated Chamber Dose (Gy)*	Δ *(%)*	*Calculated Chamber Dose (Gy)*	Δ *(%)*
FBCT	Catphan	1.635±0.067	−2.7	1.641±0.066	−2.3	1.645±0.064	−2.1
	Gammex	1.690±0.042	0.6	1.688±0.044	0.5	1.685±0.044	0.3
	SAM	1.696±0.041	0.9	1.691±0.044	0.6	1.689±0.044	0.5
	SC	1.700±0.043	1.2	1.695±0.044	0.9	1.693±0.046	0.8
CBCT	Catphan	1.599±0.062	−4.8	1.602±0.064	−4.7	1.607±0.063	−4.4
	Gammex	1.667±0.037	−0.8	1.667±0.037	−0.8	1.672±0.038	−0.5
	SAM	1.676±0.034	−0.3	1.670±0.037	−0.6	1.675±0.039	−0.3

**Table 2:** Gamma index ≤1 (3% delta dose/3 mm DTA) for absolute dose values of 2D dose distributions measured by the EDR2 film compared with dose calculation in the TPS using different HLUTs for both CT systems

*Tube Voltage*	*80 kV*	*100 kV*	*120 kV*
FBCT	Catphan	75.31%	77.99%	78.76%
	Gammex	90.59%	95.82%	98.65%
	SAM	86.73%	92.35%	97.32%
	SC	85.14%	90.00%	95.40%
CBCT	Catphan	54.27%	57.29%	64.56%
	Gammex	91.81%	93.02%	96.96%
	SAM	92.29%	94.53%	96.79%

In a clinical situation, although the radiation dose for the patient from the CBCT scanning of the Vero system can be reduced by reducing the tube voltage,^(25)^ the reduction can cause decreased treatment‐planning dose calculation accuracy if the CBCT images are utilized directly. Further, the FOV of the Vero's CBCT, which is limited to a cylinder of diameter 20 cm and length of 15 cm, could introduce truncation artifacts if the scanned object is bigger than the FOV. Hence, it is suitable only for head and neck cases.

## IV. CONCLUSIONS

The results of this investigation showed that although it is not equipped with a bowtie filter, the CBCT images of the Vero system from a complex heterogeneous phantom can be used for the dose calculation in the TPS if the system is calibrated using tissue‐equivalent materials scanned at high tube voltage (i.e., 120 kV). Further dose calculation studies on real patients can build upon the results of this work.

## ACKNOWLEDGMENTS

The presented work was performed by the second author in partial fulfillment of the requirements for obtaining the degree Dr. rer. biol. hum. at the Friedrich‐Alexander‐Universität (FAU).

## COPYRIGHT

This work is licensed under a Creative Commons Attribution 3.0 Unported License.
